# Leveraging the perioperative period to improve population health

**DOI:** 10.1186/s13741-023-00311-5

**Published:** 2023-06-05

**Authors:** Ryan Howard, Michael Englesbe

**Affiliations:** Department of Surgery, University of Michigan, 2800 Plymouth Road, Building 16, Floor 1, Ann Arbor, MI 48109 USA

## Abstract

Although surgical care has become safer, cheaper, and more efficient, it has only a modest impact on the overall health of society, which is driven primarily by health behaviors such as smoking, alcohol use, poor diet, and physical inactivity. Given the ubiquity of surgical care in the population, it represents a critical opportunity to screen for and address the health behaviors that drive premature mortality at a population level. Patients are especially receptive to behavior change around the time of surgery, and many health systems already have programs in place to address these issues. In this commentary, we present the case for integrating health behavior screening and intervention into the perioperative pathway as a novel and impactful way to improve the health of society.

Consider a hypothetical patient presenting with uncomplicated appendicitis. She is a 54-year-old woman with a body mass index of 42 kg/m^2^, poorly controlled diabetes, active tobacco use, and a high-deductible health insurance plan that has prevented her from getting regular health maintenance care. Having no major contraindication for surgery, she undergoes an uncomplicated laparoscopic appendectomy, is discharged home the next morning, and is found to have recovered completely by her 2-week follow-up visit. By every current metric, her episode of care is a success — she did not develop any complications, have a prolonged length of stay, or require any further intervention. Yet her treatable chronic conditions and her lack of access to regular healthcare will shorten her life by more than a decade (Mokdad et al. 2004). To what extent do opportunities exist within surgical care to address those problems?

Efforts to improve the value of surgical care have focused primarily on standardizing perioperative processes, controlling spending, and improving immediately measurable postoperative outcomes such as infection and readmission rates (Birkmeyer 2010; Tsai et al. 2013). While these initiatives have had substantial success and become commonplace, surgical care has at best a modest impact on the overall health of society, which is predominantly driven by health behaviors and social determinants of health. Health behaviors such as smoking, physical inactivity, unhealthy diet, and poor mental health account for nearly 50% of all premature deaths in the USA, whereas surgical care prevents less than 10% of premature mortality in this country (Schroeder 2007). While care in the perioperative period has become remarkably efficient in recent decades, opportunities to leverage this time to achieve even greater gains are still being missed. Capitalizing on health behavior change around the time of surgery has the potential to improve the value of surgical care to society and may be an impactful way to decrease death and disability in the population (Bamdad and Englesbe 2020).

The first question to be answered when considering ways to leverage surgical care to address population health problems is: why surgery? Traditionally, health behaviors and chronic conditions have been addressed within the realm of primary care. However, there are a number of challenges that threaten the effectiveness of this approach, including decreasing access to primary care physicians (PCPs) and funding cuts (Petterson et al. 2012). For example, the proportion of Americans with a PCP has decreased in recent years (Levine et al. 2020). Moreover, compared to more targeted interventions, usual primary care is often ineffective in achieving health behavior change, likely due to its routine nature and the sometimes overwhelming number of issues that need to be addressed at each visit (Asarnow et al. 2015). Conversely, surgery is a major, often transformative event in a patient’s life. As such, it serves as a powerful teachable moment (Warner 2009; Robinson et al. 2020). It has been well documented that patients undergoing a major operation are especially receptive to health behavior change. For example, while only 7% of current smokers spontaneously quit each year, over 50% of patients undergoing surgery for smoking-related diseases quit after their operation (Mustoe et al. 2020). In fact, simply undergoing any surgery, even for non-smoking-related diseases, has been shown to independently increase a smoker’s likelihood of successfully quitting compared to smokers not undergoing surgery (Shi and Warner 2010). Similar parallels exist around episodes of trauma and new major diagnoses (Ogden and Hills 2008). Just as those events often have a profound effect on the trajectory of someone’s life, the time before and after an operation represents a time when patients are particularly thoughtful and engaged in their own health outcomes.

To date, efforts to engage patients in health improvement around the time of surgery have focused primarily on the preoperative period. The practice of preoperative optimization — or “prehabilitation” — is becoming more widely accepted as an effective way to improve a patient’s overall physiologic condition before surgery (Shaughness et al. 2018). In the same way that an athlete trains for the physiologic stress of a marathon, patients can train for the physiologic stress of an operation and recovery. These programs, which usually involve increased physical activity, smoking cessation, better nutrition, and mindfulness, have been shown to reduce length of stay, decrease healthcare spending, and accelerate return to baseline functional status (Howard et al. 2019). These programs are typically limited to health gains prior to surgery, yet their success reveals the impressive level of engagement that patients exhibit around the time of surgery.

Leveraging this engagement to extend into the postoperative period and beyond could have profound effects. To that end, we believe it is time to incorporate robust health behavior screening into the perioperative pathway (Fig. 1). If ever there is a time in a patient’s life to engage them in a new trajectory for their health, it is around the time of a major surgery. Standardization of surgical care through evidence-based pathways such as enhanced recovery protocols has had a profound effect in improving short-term outcomes and lowering costs (Regenbogen et al. 2017). The effectiveness of these enhanced recovery pathways in achieving high compliance with best practices and improving outcomes could be leveraged into longitudinal health improvement efforts. Developing parallel pathways to screen for and address health behaviors such as smoking, alcohol use, physical activity, obesity, and mental health could exponentially increase the value of surgical care to patients’ overall health and to society. Such screening pathways already exist in some cases. For example, as part of trauma center verification by The American College of Surgeons, hospitals are required to employ Screening, Brief Intervention, and Referral to Treatment (SBIRT) for all trauma patients suspected of being intoxicated at presentation (Hays et al. 2020). This framework could be applied to surgical patients with any number of remediable health conditions. What’s more, virtually every health system has mechanisms in place to address these health behaviors. Rather than building a program from scratch, at many institutions, this effort may be as simple as integrating existing resources and expertise into the perioperative care pathway. For example, we described the creation of a preoperative intervention to screen for and address smoking and food insecurity that screened over 10,000 patients in its first year using existing resources and at no additional cost (Lussiez et al. 2022).

**Fig. 1:**
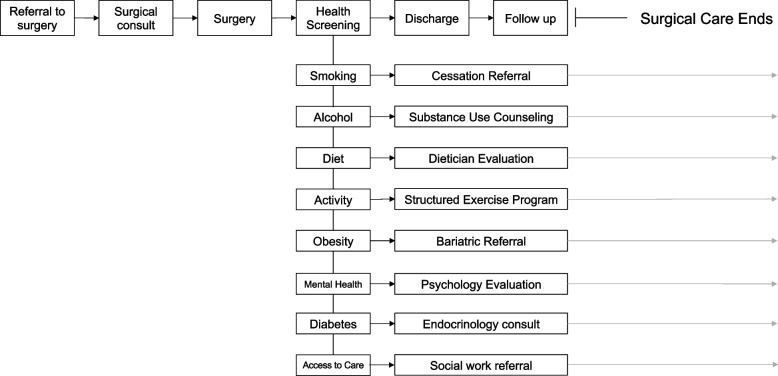
Conceptual model of incorporating health behavior screening into the perioperative care pathway

A key challenge to implementing this pathway is ensuring that it results in sustainable change. Most studies of health behavior change around the time of surgery focus on short-term (< 1 year) outcomes, and even then, recidivism is common (Howard et al. 2022). Several evidence-based strategies could be employed to increase the likelihood of sustained health improvements, such as extended contact programs that continue to engage patients longitudinally after the surgical episode, treatment tailoring that prioritizes the health improvements deemed most sustainable to each individual patient, and even skills training to help patients learn ways to combat barriers to sustained behavior change (Middleton et al. 2013). Another potential challenge will be funding these efforts, especially if sustainable improvements require long-term engagement. As mentioned above, many health systems have existing infrastructure to address these issues that could be integrated into the perioperative pathway at little to no additional cost. National insurers such as Medicaid have also shown support for programs that address chronic health conditions, and even private insurers have supported such programs in some states (Blumenthal et al. 2013). In Michigan, for example, the largest private insurer actively funds efforts directed toward increasing healthy behaviors around the time of surgery (Howard et al. 2022).

This approach may be particularly critical for patients with poor access to healthcare. A significant proportion of patients who present with surgical pathology — especially trauma or other emergent surgical problems — are underinsured and have social strain (Scott et al. 2015). The prevalence of unhealthy behaviors such as smoking or chronic illness is higher among these patients (Zhu et al. 2017). More than a quarter of trauma patients, many of whom are uninsured, have new or unmanaged medical or psychiatric comorbidities (Spruce et al. 2020). For these groups, a surgical episode may represent one of their only interactions with the healthcare system. Therefore, surgical care may be one of the only opportunities to screen for and intervene on unaddressed health problems. For these patients, surgery can serve as a gateway to care that has the potential to lengthen their lifespan. Accomplishing this, however, requires restructuring the surgical pathway to screen for and address these problems.

## Conclusions

The perioperative period is a uniquely profound time in a patient’s life. For our hypothetical patient, applying the conceptual screening pathway illustrated in Fig. 1 would identify important intervenable conditions such as smoking, obesity, diabetes, and limited access to care. Accordingly, it is not difficult to envision a scenario where prior to her discharge, she is seen by a health behavior specialist who engages her in a conversation about these issues. This evaluation could result in referrals to the hospital’s smoking cessation program, enrollment in a structured weight loss program, an inpatient endocrinology consult, and evaluation by a social worker who has resources regarding more affordable insurance and links to community resources. These simple steps could alter this patient’s overall health trajectory and lead to health improvements that last long after surgical care has ended. Embracing this opportunity would not only increase the value of surgical care to our patients and society, but is a novel, powerful way to move the needle on some of our population’s most urgent health challenges.

## Data Availability

No data was utilized for this commentary.

## Abbreviation

SBIRT: Screening, Brief Intervention, Referral to Treatment
